# Is bladder outlet obstruction rat model to induce overactive bladder (OAB) has similarity to human OAB? Research on the events in smooth muscle, collagen, interstitial cell and telocyte distribution

**DOI:** 10.1186/s13104-023-06681-9

**Published:** 2024-01-11

**Authors:** Mohamed Wishahi, Sarah Hassan, Nabawya Kamal, Mohamed Badawy, Ehab Hafiz

**Affiliations:** 1 Department of Urology, Theodor Bilharz Research Institute, P.O. 30, Warrak El-Hadar, Cairo, Imbaba, Giza, 12411 Egypt; 2 Department of Pathology and Electron microscopy, Theodor Bilharz Research Institute, Cairo, Egypt; 3 Department of Anesthesia, Theodor Bilharz Research Institute, Cairo, Egypt

**Keywords:** Overactive bladder, Bladder outlet obstruction, Experimental animal, Rats, Smooth muscles, Telocytes, Intersticial cells

## Abstract

**Background:**

Cellular and cytoskeletal events of overactive bladder (OAB) have not been sufficiently explored in human bladder due to different limitations. Bladder outlet obstruction (BOO) had been induced in different animal models with different methods to induce (OAB). Similarity of the animal models of BOO to the human OAB is postulated but has not been confirmed. The interstitial cells of Cajal (ICCs), and telocytes (TCs) are an important players in smooth muscles conductivity, they had not been well investigated in the previous BOO models. Objectives are to investigate the morphological pattern of cellular, cytoskeleton and telocytes distribution in BOO rat model and to match the events in two time periods and compare it to the findings in real-world human OAB.

**Methods:**

Female Sprague-Dawley rats (*Rattus norvegicus)* were randomly divided into: sham (n = 10), BOO 6 W (n = 10), BOO 8 W (n = 10). Operative procedure to Induce BOO was done under anesthesia with intraperitoneal Ketamine administration. The Effect of induction of BOO was evaluated after 6 and 8 weeks. The rats were anesthetized, and the urinary bladder was removed, while the rat was unconscious under anaesthesia it was transferred to the inhalation anaesthesia cage for euthanasia, rats were sacrificed under light anesthesia using isoflurane. Care of animals, surgical procedure, and euthanasia adhered to Guide for the Care and Use of Laboratory Animals, and AVMA Guidelines for the Euthanasia of Animals. The retrieved bladder was processed for examination with histopathology, immunohistochemistry (IHC), and transmission electron microscopy (EM).

**Results:**

Histological examination of the bladder shows thinner urothelium, condensation of collagen between muscle bundles. IHC with c-kit shows the excess distribution of ICCs between smooth muscle bundles. EM shows frequent distribution of TCs that were situated between collagen fibers. Finings in BOO 6 W group and BOO 8 W group were comparable.

**Conclusion:**

The animal model study demonstrated increased collagen/ smooth muscle ratio, high intensity of ICCs and presence of TCs. Findings show that a minimally invasive procedure to induce BOO in rats had resulted in an OAB that has morphological changes that were stable in 6 & 8 weeks. We demonstrated the distribution of TCs and ICCs in the rat animal model and defined them. The population of TCs in the BOO rat model is described for the first time, suggests that the TCs and ICCs may contribute to the pathophysiology of OAB. Similarity of animal model to human events OAB was demonstrated. These findings warrant further study to define the role of TCs in OAB.

**Clinical trial registry:**

The study does not require a clinical trial registration; it is an experimental animal study in basic science and does not include human subjects.

## Introduction

Overactive bladder (OAB) is a condition that affects both children and adults, leading to lower urinary tract symptoms. This occurs to poor bladder compliance and detrusor overactivity, which causes involuntary detrusor contractions and high detrusor pressures, ultimately affecting the upper urinary tract. While there are various treatment options available for OAB, recent treatments, and pharmaceutical manipulations suggest an incomplete understanding of the pathophysiology and morphological changes of OAB [1].

Previous studies on OAB in animal models have primarily focused on functional characterization, molecular mechanisms, and drug administration, but these models have lacked similarity to human bladder events. A bladder outlet obstruction (BOO) animal model that was implemented surgically to replicate outlet obstruction in human was based on the induction of a narrow urethral lumen via a ligature around the urethra or the bladder neck. Surgical induction of lower urinary tract dysfunctions has been induced in numerous animal species, such as rabbits, rats, guinea pigs, mice, pigs, and dogs, of both sexes [2]. These surgical procedures have led to morphological and functional changes in the bladder, including collagen and smooth muscle distribution, detrusor muscle hypertrophy, collagen deposition, interstitial cell density, urothelial changes, reduced bladder capacity, and changes in neurotransmitters [2, 3].

Kang et al. and Jin et al. have described an open surgical procedure to induce BOO, involving an abdominal incision to visualize the proximal urethra and bladder [3, 4]. A variant technique was described by Dunton et al. and others [5–7].

The open surgical techniques used to induce BOO have been associated with a high incidence of complications, longer operation time, and inflammation around the urethral ligation that results in increased urethral obstruction. The incision and closure of the abdominal muscle layer and skin have also been reported to lead to fibrosis. These events do not occur in human BOO and may cause deviations in the accuracy and similarity of this model to human events [8].

Several minimally invasive techniques have been described to induce BOO, including the injection of the urethral orifice with hyaluronic acid [9]. Monopolar cauterization of the urethra and perineal approach has been applied to induce BOO [10].

Kim et al. described a minimally invasive procedure to induce OAB that avoids the side events and complications of open procedures. This procedure involved passing a metal rod through the urethra and applying a suture in the peri-urethral tissues in the skin area [11].

Previous studies on OAB in animal models have observed changes in pressure parameters during the filling and voiding phases, followed by increased residual volume and decreased micturition volume. Decompensated bladders have been observed due to residual urine during the later phase. The subsequent pathophysiological changes in the bladder were classified as overactive bladder, decompensated bladder, and fibrotic bladder. These changes were observed starting from 4 weeks and progressing through 8 weeks, and up to 16 weeks, respectively [6, 10–13].

Morphological changes in OAB in these animal models showed an increased amount of collagen tissue, changes in smooth muscles, and an increased thickness of the bladder wall. It has been suggested that the increased connective tissue in OAB may play a relevant role in achieving normal bladder function. Uvelius et al. demonstrated an increased total amount of bladder collagen during obstruction, which decreased after de-obstruction. They proposed that these changes are due to increased functional demands placed on the bladder by BOO, such as an increase in tension [14].

In both rat and guinea pig models of OAB, there have been findings that suggest the potential involvement of interstitial cells of Cajal (ICCs) in the pathophysiology of the condition. Specifically, in female rats, an increase in the population of ICCs was observed in the submucosa and muscle layers, indicating a possible role in functional changes [15]. Similarly, Kubota et al. demonstrated the altered distribution of ICCs in guinea pig bladder following BOO [16].

A recent study has shed light on the significance of telocytes, a specific type of interstitial cells, in multiple organs [17]. The role played by telocytes in various organs in health and disease has drawn more attention recently, and it has been demonstrated that telocytes (TCs) play a significant role in the urinary system [18–21].

Recent study on the human urinary bladder with OAB demonstrated the distribution of ICCs and TCs with an increased collagen deposition [20].

The aim of this study is to create a minimally invasive experimental model in female rats to induce BOO to investigate the changes in smooth muscles, collagen, ICCs, and the expression of TCs. The study is reporting the cellular and cytoskeleton changes in 2 time periods of 6 and 8 weeks after inducing BOO and correlated to the control group. Changes will be compared to the events in human OAB that was reported before.

## Materials and methods

### Animal model

#### Ethics approval

The experimental protocol and the study were done according to the requirement of National Research Council 2011 “Guide for the Care and Use of Laboratory Animals: Eighth Edition. Washington, DC: The National Academies Press, and approved by the Research Ethics Committee of Theodor Bilharz research institute for the conduct of the animal experiments [PT 639].

### Animal

Thirty female Sprague–Dawley rats (*Rattus norvegicus*) weighting 220 ± 10 g were obtained from and kept in the animal house of Theodor Bilharz Research Institute- Cairo, Egypt, they were housed in a group of two per cage in polypropylene cage at room temperature of 20–25 °C, and had free access to standard chow diet and water with a 12-hour light/ 12-hours dark light cycle. Animals were kept on the normal chow and water with a 12-hour day/night light cycle for 1 week to adapt to the new environment.

Care of animal, surgical procedure, and euthanasia was conducted in adherence to the Guide for the Care and Use of Laboratory Animals (Eighth edition) of the National Institutes of Health, complied with the ARRIVE guidelines, and AVMA Guidelines for the Euthanasia of Animals, and approved by the Research Ethics Committee of TBRI for the conduct of animal experiments [PT 639].

### Experimental design

This study is designed on independent three groups, rats were randomly divided into three groups: Sham (n = 10), BOO for 6 weeks (BOO 6 W, n = 10), BOO for 8 weeks (BOO 8 W, n = 10). After induction of BOO for 6 weeks (BOO 6 W, n = 10), and 8 weeks (BOO 8 W, n = 10), and the sham group (n = 10), the urinary bladder was retrieved and examined to detect changes in the cellular and microskeleton elements.

### Surgical procedures

#### BOO procedure

**Fig. 1:**
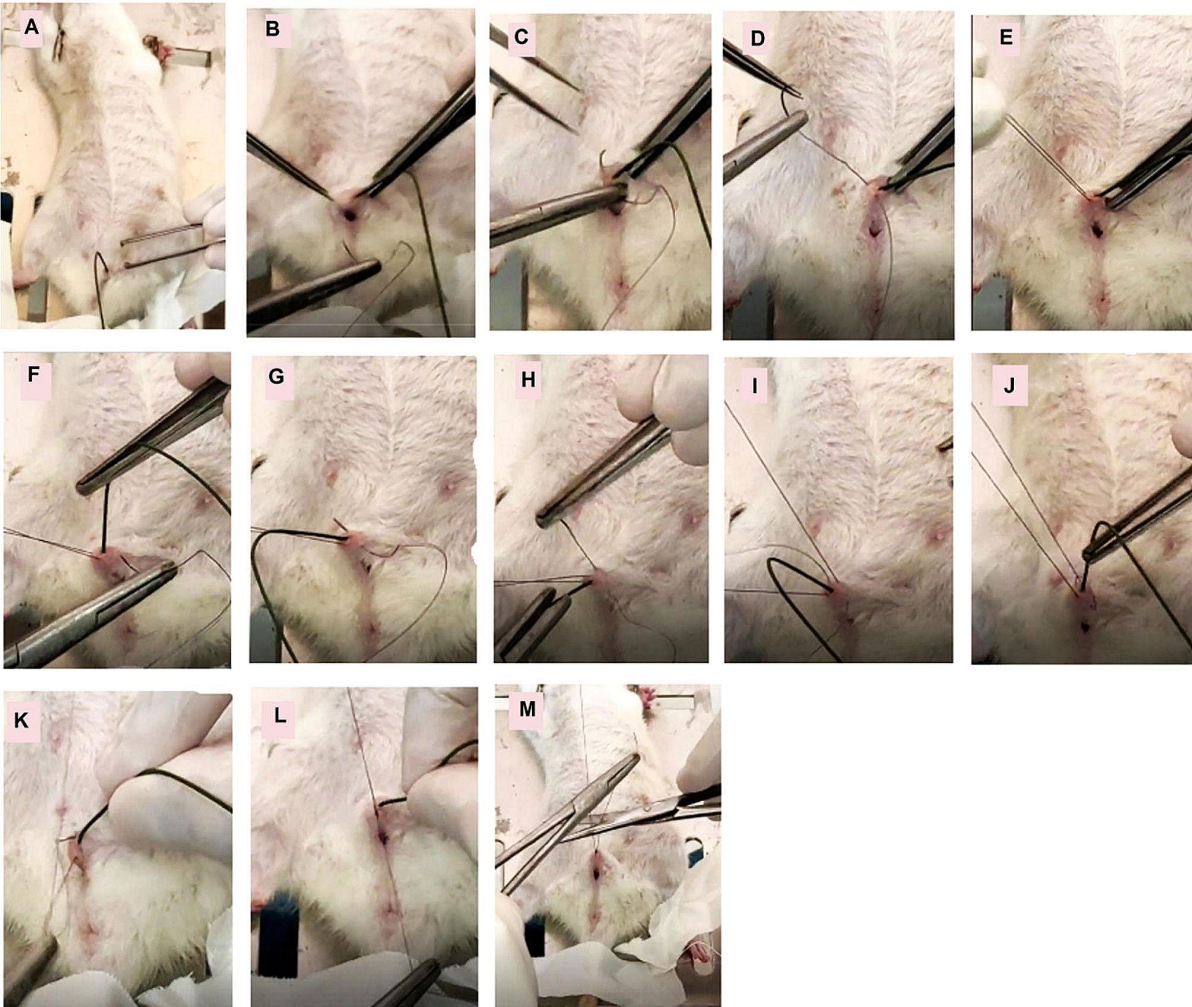
Minimally invasive procedure for induction of partial urethral obstruction to induce overactive bladder. (**A**) The female rat is under anesthesia, a floppy guidewire of 0,038 inches in diameter is passed to the bladder. (**B**, **C**) Curved needle with 4/0 proline thread is passed to the para-urethral tissues in the right side, but not punching the urethra. (**D**) The included tissues are raised up to illustrate that the suture is away from the vagina and the guidewire is in place in the urethra. (**E**) Tissues are raised to show that the suture is away from the vagina. (**F**, **G**, **H**, **I**) Needle is passing into the left paraurethral tissues while the guidewire is in the urethra. (**J**) The suture had passed in the right and left paraurethral tissues. (**K**, **L**) Making a tight suture around the urethra while the guidewire is in place inside the urethra. (**M**) Removal of the guidewire and removing the excess parts of the thread

The rats were anaesthetized with ketamine 10 mg/100 g body weight via intraperitoneal injection, rats were placed in a supine position, the skin of the lower abdomen and vagina were disinfected with 70% alcohol, a sterile floppy guidewire size 0.038 inches in diameter (0.965 mm) (Amecath Medical Technologies US Inc., Wilmington County, Delaware, USA) is placed in the urethra and advanced to the bladder, a 4 − 0 silk suture over curved needle (ETHICON, Inc. Somerville, NJ. USA) is placed around both sides the urethra, the suture is secured over the guide wire in the urethra, the guidewire is removed, leaving the urethra partially obstructed (Fig. 1). After recovery from anesthesia, rats were returned to their home cages. They had free access to food and water. Identical procedures were performed without the urethral tie being tied for the sham-control group. Rats were returned to their sterile cages, with free access to food and water. All survived except 2, rats were kept on normal chow.

#### Retrieval of the urinary bladder

At the end of 6 weeks (n = 10), and 8 weeks (n = 9) of the guinea-pig BOO and Sham group (n = 9), each rat is taken from its cage by the animal-care personnel weighted with a laboratory scale and transported to the operation room of the experimental animal house, the rat is handled gently with the veterinary doctor, an expert anaesthetist induced anaesthesia with ketamine 10 mg/100 g body weight via intraperitoneal injection. The anaesthetist determines the status of deep anaesthesia, a lower abdominal incision of 2 cm is made. The urinary bladder and the bladder neck region were examined for signs of adhesion or fibrosis. The urinary bladder is removed, the incision is closed with one suture, and the procedure lasts for 3 min.

#### Animal euthanasia

While the rat is unconscious under anaesthesia it is transferred to the inhalation anesthesia cage for euthanasia. Rats were sacrificed under light anesthesia using isoflurane (Forane®, Baxter, UK) inhalation. The anaesthetist Adjust the isofurane flow rate or concentration to 5% or greater and continue isoflurane exposure until one minute after breathing stops. The anesthetist confirms euthanasia with: lack of a heartbeat, lack of respiration, lack of corneal reflex, and the presence of rigor mortis. We adhered to AVMA Guidelines for the Euthanasia of Animals: 2020 (Edition AVMA Guidelines for the Euthanasia of Animals: 2020 Edition. AVMA.org: 2020. Last Reviewed by the IACUC 1/11/2023), Frost et al., 2020, and Shomer et al. 2020 [22–24].

The urinary bladder was grossly examined, weighted, and processed for examination with histopathology, immunohistochemistry (IHC), and transmission electron microscopy (EM).

#### Gross examination

Each rat was weighted, and the bladder was weighted separately.

#### Bladder hypertrophy

In the 3 groups, each rat was weighted, and the retrieved bladder was weighted separately.

#### Histological examination

The bladder was examined grossly for the wall thickness. Samples from the bladder were divided into two parts, one for histopathology and the other for electron microscopy examinations. Tissues to be examined for histopathology were immediately fixed in a 10% formalin solution, processed, and embedded in paraffin. Sections of 4 microns thickness were stained with H&E stain for histological evaluation; Masson’s trichrome staining to assess the smooth muscles to collagen ratio.

### Immunohistochemical examinations for c-Kit positive interstitial cells

Immunohistochemistry was performed on 5 microns thick sections of formalin-fixed and paraffin-embedded tissue samples. Sections were picked on charged glass slides and deparaffinised, hydrated, then treated for antigen retrieval at a high pH (pH 8) using an automated immunostainer (Dako, Denmark). Rabbit polyclonal anti-CD117 (c-kit) antibodies (CD 117, Cat. No. A4502, Dako, Denmark) at dilution 1:200 was used to label the interstitial cells. Goat anti-rabbit biotinylated immunoglobulins/HRP (Cat. No. P0448, Dako, Denmark) were used at dilution 1:300. Streptavidin–biotin–peroxidase complex and peroxidase-DAB (3,3’diaminobenzidine) (Dako, Denmark) detection method was preformed according to the manufacturer’s instructions. Sections were counterstained with Mayer’s haematoxylin. Positive and negative control slides were included in each run. As a negative control, a tissue section was processed as described but with the primary antibody omitted. Minimal of 10 fields were examined in each section at different magnification, and a semiquantitative analysis of distribution of ICCs. The pathologist was blended for the sample origin being BOO or sham-normal and 6 or 8 weeks duration of BOO.

### Transmission Electron microscopy examination

Immediately following surgical excision of the rat bladder, the bladder specimens were cut into 1 × 1 mm pieces, fixed in 2.5% glutaraldehyde in cacodylate buffer for 2 h at 4^o^C. The tissues were then washed in cacodylate-sucrose buffer and post fixed for 1 h at 4^o^C in 2% osmium tetroxide. After dehydration in graded ethanol, the samples were impregnated in Epon 812 substitute (EMBed-812 Kit, Electron Microscopy Science, USA) at room temperature, and polymerised at 60 °C for 48 h. Semi-thin sections were cut, stained with methylene blue-azure II, and examined by light microscopy to choose the region of interest for ultrathin sectioning. The ultrathin sections were then prepared using an Ultracut R ultramicrotome (Leica, Vienna, Austria), double stained with uranyl acetate and lead citrate. Cellular ultrastructural morphological characterisation was examined at 80 kV with a Jeol TEM Jem-1400 electron microscope (Jeol, Japan). Identification and differentiation of telocytes and Cajal was done according to the description in previous works. We examined each case and counted telocytes and/or telopodes (TPs) in 3 examined TEM fields.

### Statistical analysis

GraphPad Prism (version 8.0.0, USA) was used to analyse the data. All results were presented as mean ± standard deviation (SD). One-way analysis of variance (ANOVA) was used, followed by the Tukey multiple-comparison *post hoc* test to compare between the different groups. A *p* value < 0.05 was considered statistically significant.

## Results

### Animals

The three groups of rats had normal activities for access to food and water, and voiding. The site of the urethral ligation was healthy with no evidence of infection, the tied suture was in place. Laparotomy revealed no adhesions or fibrosis in the abdominal wall, around the bladder, and in the bladder neck region.

### Bladder hypertrophy

We measured the bladder weight to evaluate whether it had increased in the BOO animals compared to the sham group. The bladder weight in the control group was 110 ± 7 mg, bladder weight in BOO 6 W; BOO 6 W was 130 ± 15 g. Body weight did not differ between BOO 6 W, BOO 8 W and sham group. The bladder/body weight ratio was also comparable between the 3 groups.

### Anatomical gross inspection

Examination of urinary bladder of BOO 6 W and BOO 8 W groups showed bladder wall thickening compared the sham group.

#### Histopathology of urinary bladder of OAB and sham-control

**Fig. 2:**
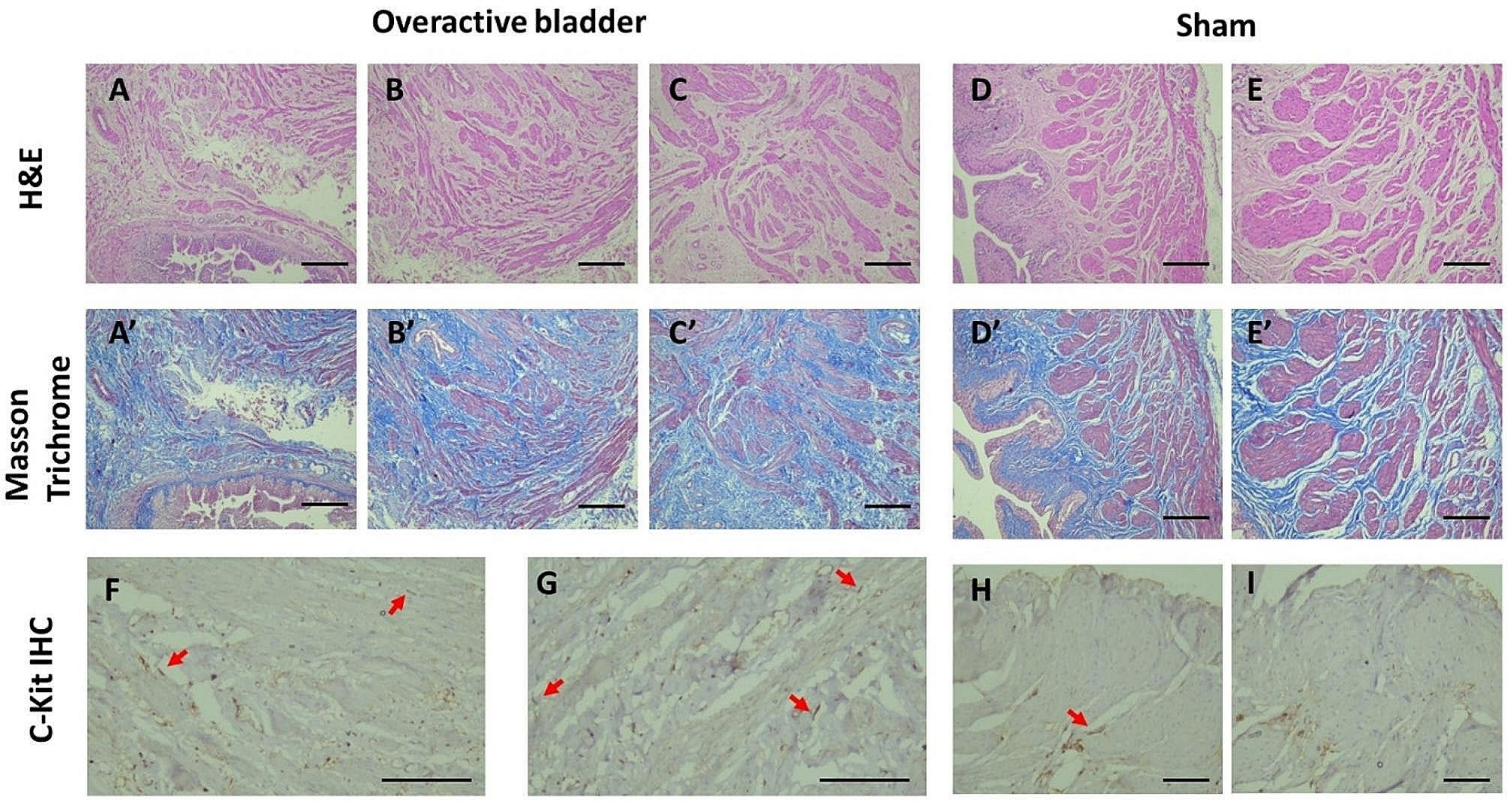
Histopathology and immunohistochemistry of overactive bladder in BOO 6 W, BOO 8 W, and sham groups of female rat animal model. Upper horizontal column in the panel: Hematoxylin and eosin (H&E) staining of OAB and sham control of female rat urinary bladder (**ABCDD’**): Representative sections of urinary bladder from OAB at 6 & 8 weeks (**A**, **B**, **C**), and control (**D**,**D’**), the urothelium, muscle bundles in muscle layer are well arranged and condensed with few interlacing collagens, contrary to bladder wall of OAB where the urothelium is less in thickness, muscle bundles are separated with condensation of collagen. Scale bar = 50 μm. Middle horizontal column in the panel: Masson’s trichrome staining for OAB (**A**^’^**B**’**C**’**D**’) and control (**EE’**) of female rat urinary bladder, respectively, stained slides in this column are representative of the tissues stained with H&E in the upper column. Urinary bladder smooth muscle cells were stained red and collagen fibers were stained blue. Collages were density interlacing the muscle bundles in OAB group, the control group showed normal distribution of collagen in between muscle bundles. Scale bar = 50 μm. Lower horizontal column in the panel: Immunohistochemical staining of OAB and sham control (**FGHI**): Distribution of c-Kit-immunoreactive interstitial cells in detrusor smooth muscle. C-Kit positive immunoreactive cells are detected along the muscle bundles and smooth muscle cells surrounding the detrusor muscle layers. In overactive bladders, immunoreactivity against c-kit was highly expressed in OAB after 6 & 8 weeks of BOO (**G**, **F**- small red arrows) compared with control where it was not or seldom detected (**H**, **I**- small red arrow). Scale bar = 50 μm

Sections of the bladder wall of BOO 6 W, BOO 8 W, and sham group were examined with H&E staining and a corresponding tissue section were examined with Masson’s trichrome staining. OAB showed that urothelium is thinner, and the muscle bundles are separated with condensation of collagen. In the sham group, the urothelium was normal, muscle bundles and muscle layer are well arranged and has few interlacing collagens. Findings at 6 weeks of BOO were similar to that of 8 weeks. Collagens were density interlacing the muscle bundles in OAB group, with a collagen to smooth muscle ratio of 2, the sham group showed normal distribution of collagen in between muscle bundles (Fig. 2).

#### Immunohistochemistry

Immunohistochemical staining of OAB and sham-control with c-Kit to examine the distribution of interstitial cells in relation to detrusor smooth muscle. C-Kit positive immunoreactive cells were detected along the muscle bundles in between smooth muscle cells in the detrusor muscle layers and were highly expressed and densely distributed in the two groups BOO 6 W & BOO 8 W compared with sham group where it was not or seldom detected (Fig. 2).

#### Transmission electron microscopy

**Fig. 3:**
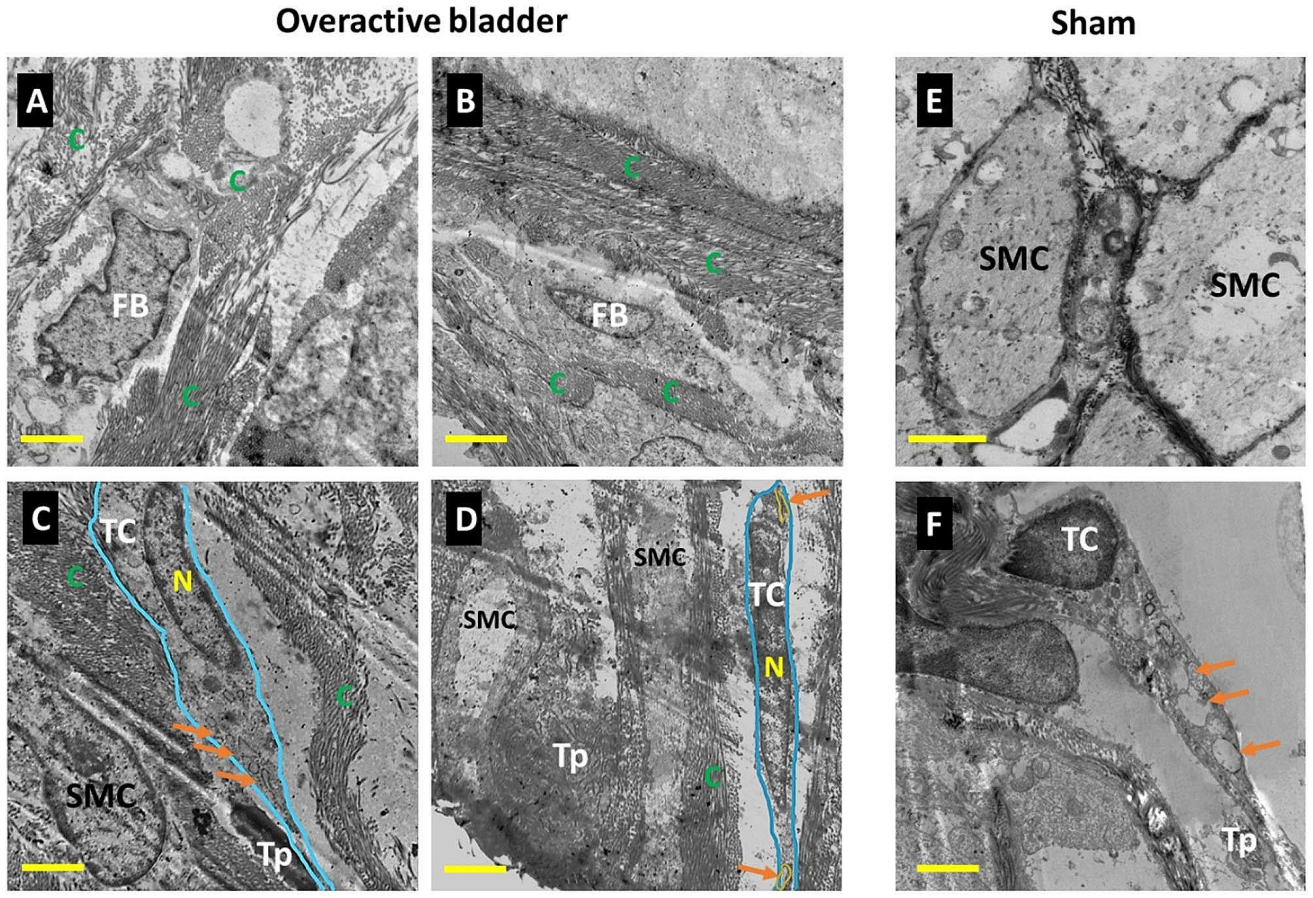
Transmission electron microscopy of overactive bladder in BOO 6 W, BOO 8 W, and sham groups of female rat animal model. (**A**) Overactive bladder: Condensation of collagen (C) surrounding a fibroblast cell (Fb). Scale bar 2 μm. (**B**) Overactive bladder: Condensation of collagen (C) and absent telocyte. Scale bar 2 μm. (**C**) Overactive bladder: Smooth muscle cell (SMC) adjacent to bundle of collagen (C). Telocyte (TC) (marked boundaries with blue) having its nucleus (N) and long slender cytoplasm of Telopode (Tp) containing a vesicle pointed with small orange arrows and is situated between condensation of collagen. Scale bar 1 μm. (**D**) Overactive bladder: Smooth muscle cell (SMC) with many telopodes extensions (Tp) in between. Telocyte (TC) (marked boundaries with blue) containing a nucleus (N) and long cylindrical telopodes (Tp) that contains a vesicle (V) pointed with small orange arrows and is situated between condensation of collagen. Scale bar 1 μm. (**E**) Normal control. Smooth muscle cells (SMC) are orderly arranged with close cell to cell contact with minimal collagen in the interstitium. Scale bar 2 μm (**F**) Normal control: Telocyte (TC), nucleus (N), telopodes (Tp) contain many vesicles pointed with small orange arrows. Scale bar 1 μm

EM examination of OAB demonstrated the smooth muscle cell in adjacent to condensation of collagen bundles within the detrusor layer. Telocytes with their distinct nuclei and the long cylindrical tapered varicose-like cytoplasmic processes of telopodes (Tps) that contain numerous vesicles were situated between condensations of collagen fibres. Fibroblasts were detected in some cases; other cases did not demonstrate the presence of TCs. EM of sham-normal control showed that smooth muscle cells are orderly arranged with regular outline and close cell-to-cell contact with minimal collagen in the interstitium. While those of the OAB, showed invaginations of the cell membrane with more abundant extracellular matrix. Telocyte were demonstrated in the control group (Fig. 3).

**Fig. 4:**
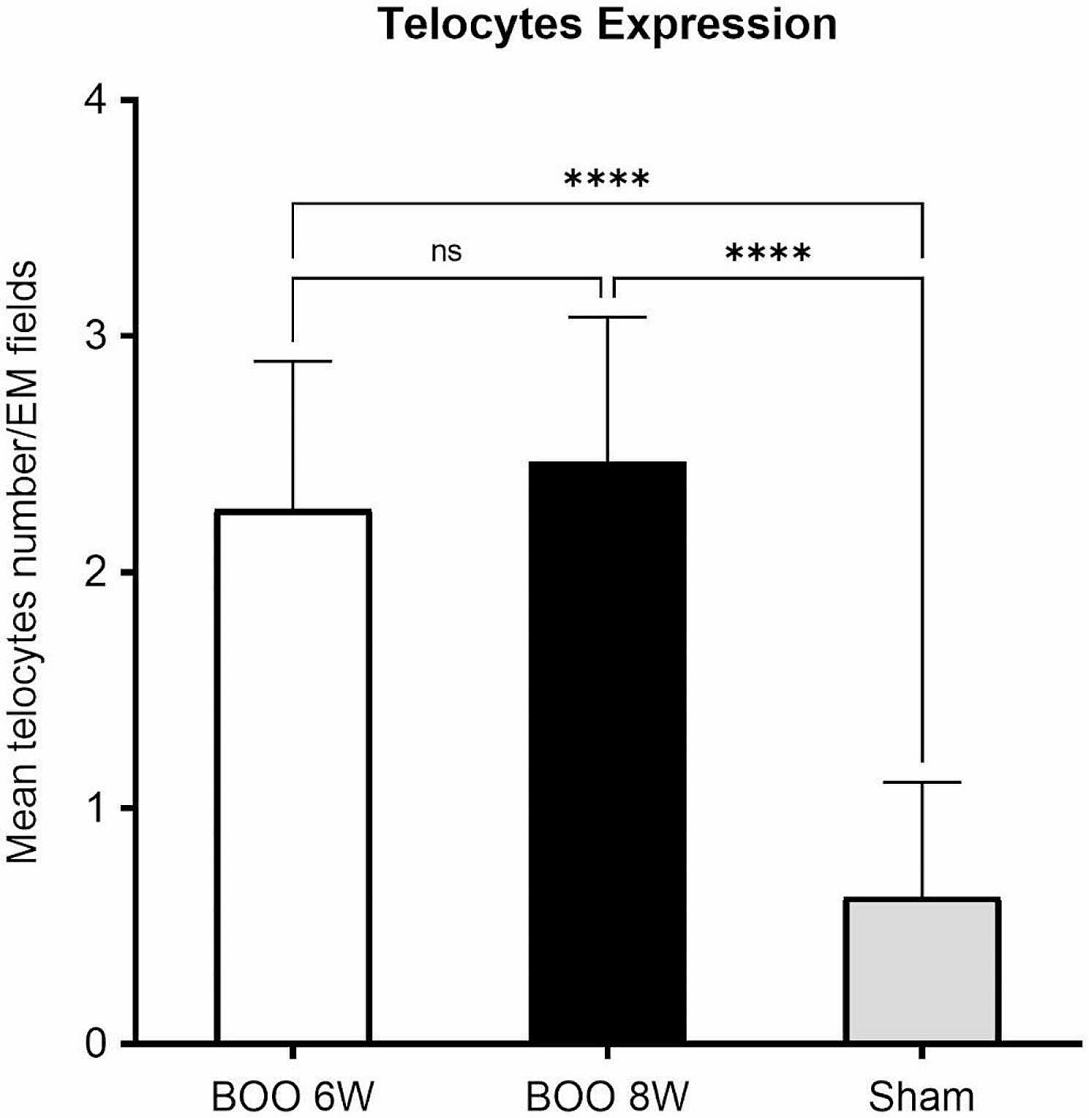
Relative quantitative distribution of TCs/TEM fields. Results are expressed as mean ± SD, ****p < 0.001. BOO 6w (n = 10), Boo 8w (n = 10) and Sham (n = 8). Abbreviations: BOO (bladder outlet obstruction)

Distribution of TCs and Tps was detected in BOO 6 W and BOO 8 W of OAB after 6 & 8 weeks of BOO with a mean of 2.27 ± 0.7 cells/TEM fields and a mean of 2.47 ± 0.7 cells/TEM fields respectively, compared to 0.62 ± 0.4 cells/TEM fields of the sham group (p < 0.001) (Fig. 4).

## Discussion

Results indicate that induction of BOO using minimally invasive technique inspired by the descried procedure of Kim et al. [11]. In the present closed technique, the abdomen and the bladder were not opened, and the perivesical and periurethral tissues were not dissected, consequently avoided tissue manipulation that occurred in the open surgical techniques for induction of BOO animal model that had been reported to be associated with a high incidence of complications [2, 8]. In the present technique there was no encountered post-operative complication. Open technique reported longer operation time, while our technique is done in 5 min. An explanation on the success of the procedure and absence of complication and fast procedure is the use of a sterile guidewire to be inserted in the urethra which is the same one that we are using in the minimally invasive procedure in urology.

Earlier studies have shown that muscle mass increases in response to BOO in the experimental model [6]. In our current study, we have demonstrated an increased in bladder weight, muscle hypertrophy, and the predominance collagen content in which is consistent with the findings in previous reports [2, 14].

Few studies demonstrated the presence of ICCs in the OAB of animal model. It is suggested that the pathophysiology of OAB may be attributed to different elements which include the smooth muscle cells, nerves, and ICCs. Kim et al. demonstrated the distribution of ICCs in the bladders of an ovariectomized female Sprague-Dawley rats that developed OAB. ICCs were distributed in the urothelial and muscle layers. They suggested that ICCs is playing a role in detrusor overactivity and normality of bladder function [15].

Kubota et al. investigated the distribution of interstitial cells (ICs) in the guinea pig bladder after BOO, they validated the bladder overactivity of BOO animals with urodynamic studies. Immunohistochemical analyses for c-Kit was performed on both BOO and control bladders. They found that 2 weeks after induction BOO there was an increase of bladder wall thickness. The suburothelial and subserosal connective tissue layers was increased. C-kit immunoreactive ICs was demonstrated in subserosal layers and was altered in suburotherial layer. These results demonstrated the increased population of ICs in the BOO guinea pig model and suggest that the altered distribution of ICs may contribute to the pathophysiology of bladder overactivity. The guineapig bladder model of BOO showed the distribution of ICCs similar to our findings in our rat model. These finding supports the suggestion that the ICCs and TCs has a role in the etiopathology of OAB [16].

Recently, a study investigated the cellular and cytoskeleton in human overactive bladder. Tissues were examined for light microscopy, IHC, and EM. Histopathology elicited that the muscle/collagen ratio was 0.5/3, IHC revealed c-kit positive cells of ICCs. Electron microscopy examination showed the distribution of telocytes [20].

The present study demonstrates the excess distribution of TCs in BOO model of Exp-6 & Exp-8 groups is comparable to the control group. The presence of ICCs and TCs in the BOO in the rat model is similar to the findings in the human OAB, furthermore, the distribution of smooth muscles and collagen in the BOO model is similar to that in the human OAB [20].

The changes in the cellular and cytoskeleton of OAB rat model at 6week and 8 weeks confirms the clinical findings in real-world OAB in benign prostatic hyperplasia, urethral strictures, urethral stones, infravesical partial obstruction in women, and children [1] In real-world clinical presentation of lower urinary tract symptoms secondary to OAB, the bladder changes are stable and seldom to become decompensated or become fibrotic. These clinical findings were observed in the rat model where changes were stable after 8 weeks of obstruction.

Based on our findings, we hypothesized that the excess distribution of TCs in BOO rat model and human OAB may serve as an initiating factor for detrusor overactivity, enabling the communication between smooth muscle cells to overcome BOO, furthermore, it would have a role in the regenerative process of damaged smooth muscle cells caused by obstruction, and they may contribute in the regenerative events that occur in the obstructed bladder once the obstruction is relieved.

Previous studies have indicated that the onset of OAB typically occurs around 4–6 weeks after induction, followed by bladder decompensating and subsequent fibrosis [6]. In our study, we observed the changes associated with BOO at 6th and 8th weeks. The reported changes in both groups were close to each other, this observation indicates that the cellular and cytoskeleton changes stabilise after the 6th week.

Based on our findings, we hypothesized that the overexpression of TCs in BOO and human OAB may serve as an initiating factor for detrusor overactivity, enabling the communication between smooth muscle cells to overcome BOO, has role in the regenerative process of damaged smooth muscle cells caused by obstruction, and they may contribute in the regenerative events that occur in the obstructed bladder once the obstruction is relieved.

## Conclusion

The present study showed that a minimally invasive procedure for induction of BOO to induce OAB through an external partial urethral obstruction for 6 & 8 weeks resulted in a similar events that has similarities that developed in the human OAB in regard to excess collagen content, the distribution of telocytes and Cajal cells. The similarity of the minimally invasive induction of BOO model to simulate OAB in human indicates confidentiality in future research on OAB.

### Limitation

There is some limitation of the present of the study: 1-The voiding pattern of the rat model of BOO was not recorded; 2- The metabolic changes in the bladder after BOO were not studied; 3- The morphological changes following subsequent relieving obstruction in this model was not investigated. In our study we aimed at demonstrating the validity of a minimally invasive technique to induce OAB in an experimental animal, second to investigate the distribution of telocytes in the OAB that may open a new horizon in future personalised medicine.

## Data Availability

The dataset generated and/ or analyzed during the current study are presented in the publication.

## Abbreviations

BOO: Bladder outlet obstruction
OAB: Overactive bladder
ICCs: Interstitial cells of Cajal
TCs: Telocytes
Tps: Telopodes
EM: Electron microscopy
HE: Haematoxylin and eosin
IHC: Immunohistochemestry
BOO 6W: experimental model of rat bladder outlet obstruction that was maintained for 6 weeks
BOO 8W: experimental model of rat bladder outlet obstruction that was maintained for 8 weeks
